# Landscape of epigenetically regulated lncRNAs and DNA methylation in smokers with lung adenocarcinoma

**DOI:** 10.1371/journal.pone.0247928

**Published:** 2021-03-08

**Authors:** Jiyoon Jung, Yoo Jin Lee, Chul Hwan Kim, Sangjeong Ahn

**Affiliations:** 1 Department of Pathology, International St. Mary’s Hospital, Catholic Kwandong University College of Medicine, Incheon, Republic of Korea; 2 Department of Pathology, Anam Hospital, Korea University College of Medicine, Seoul, Republic of Korea; Chinese University of Hong Kong, HONG KONG

## Abstract

In this study, we identified long non-coding RNAs (lncRNAs) associated with DNA methylation in lung adenocarcinoma (LUAD) using clinical and methylation/expression data from 184 qualified LUAD tissue samples and 21 normal lung-tissue samples from The Cancer Genome Atlas (TCGA). We identified 1865 differentially expressed genes that correlated negatively with the methylation profiles of normal lung tissues, never-smoker LUAD tissues and smoker LUAD tissues, while 1079 differentially expressed lncRNAs were identified using the same criteria. These transcripts were integrated using ingenuity pathway analysis to determine significant pathways directly related to cancer, suggesting that lncRNAs play a crucial role in carcinogenesis. When comparing normal lung tissues and smoker LUAD tissues, 86 candidate genes were identified, including six lncRNAs. Of the 43 candidate genes revealed by comparing never-smoker LUAD tissues and smoker LUAD tissues, 13 were also different when compared to normal lung tissues. We then investigated the expression of these genes using the Gene Expression of Normal and Tumor Tissues (GENT) and Methylation and Expression Database of Normal and Tumor Tissues (MENT) databases. We observed an inverse correlation between the expression of 13 genes in normal lung tissues and smoker LUAD tissues, and the expression of five genes between the never-smoker and smoker LUAD tissues. These findings were further validated in clinical specimens using bisulfite sequencing, revealing that *AGR2*, *AURKB*, *FOXP3*, and *HMGA1* displayed borderline differences in methylation. Finally, we explored the functional connections between DNA methylation, lncRNAs, and gene expression to identify possible targets that may contribute toward the pathogenesis of cigarette smoking-associated LUAD. Together, our findings suggested that differentially expressed lncRNAs and their target transcripts could serve as potential biomarkers for LUAD.

## Introduction

Lung cancer is the leading cause of cancer-related deaths worldwide [1]. Cigarette smoke (CS) exposure is known to affect epigenetic regulation [2,3] and has been established as a critical factor in the development of lung cancer [4–8]. Epigenetic modifications include DNA methylation, histone modifications, and the modulation of non-coding RNAs [9–11], among which CS is widely known to alter DNA methylation and thereby cause lung cancer [12–15].

Long non-coding RNAs (lncRNAs) are transcripts of over 200 nucleotides in length that lack or have a limited protein-coding potential [16,17]. Recently, lncRNAs have received considerable attention as epigenetic regulators [18,19], with an increasing number of studies implicating lncRNAs in several functions related to carcinogenesis and the progression of lung cancer [20–24]. Until now, only a limited number of CS-associated lncRNAs have been reported in lung cancer, namely, *Smoke and Cancer-associated LncRNA–1* (*SCAL1*), *HOX Transcript Antisense RNA* (*HOTAIR*), *H19*, and *Metastasis-Associated Lung Adenocarcinoma Transcript 1* (*MALAT1*) [25–30]. Since these lncRNAs have not been explored thoroughly with CS-related epigenetic regulation, further studies are required to identify additional lncRNAs and their possible roles in epigenetic regulation.

In this study, we aimed to investigate the relationship between DNA methylation and lncRNA expression in lung adenocarcinoma (LUAD) and thereby elucidate the landscape of lncRNAs associated with DNA methylation-mediated regulation in smokers.

## Materials and methods

### Datasets

Level 3 expression and matched DNA methylation data for LUAD were downloaded from The Cancer Genome Atlas (TCGA) data portal (https://portal.gdc.cancer.gov/) in January 2017. Only patients with available smoking history with their clinical information were included, amounting to 184 LUAD and 21 normal lung tissues with fully characterized expression and matched DNA methylation data assayed using Illumina Infinium Human Methylation 450K.

For the validation cohort, 76 samples were collected from patients with LUAD who had undergone surgery at the Korean University Medical Center between 2010 and 2013 (Seoul, Korea). Samples were fixed and processed according to clinical standard operating procedures. The specimens and data used in this study were provided by Korea University Anam Hospital and approved by the appropriate Institutional Review Board (2014AN0393).

### Differential lncRNA expression and DNA methylation

**Fig 1:**
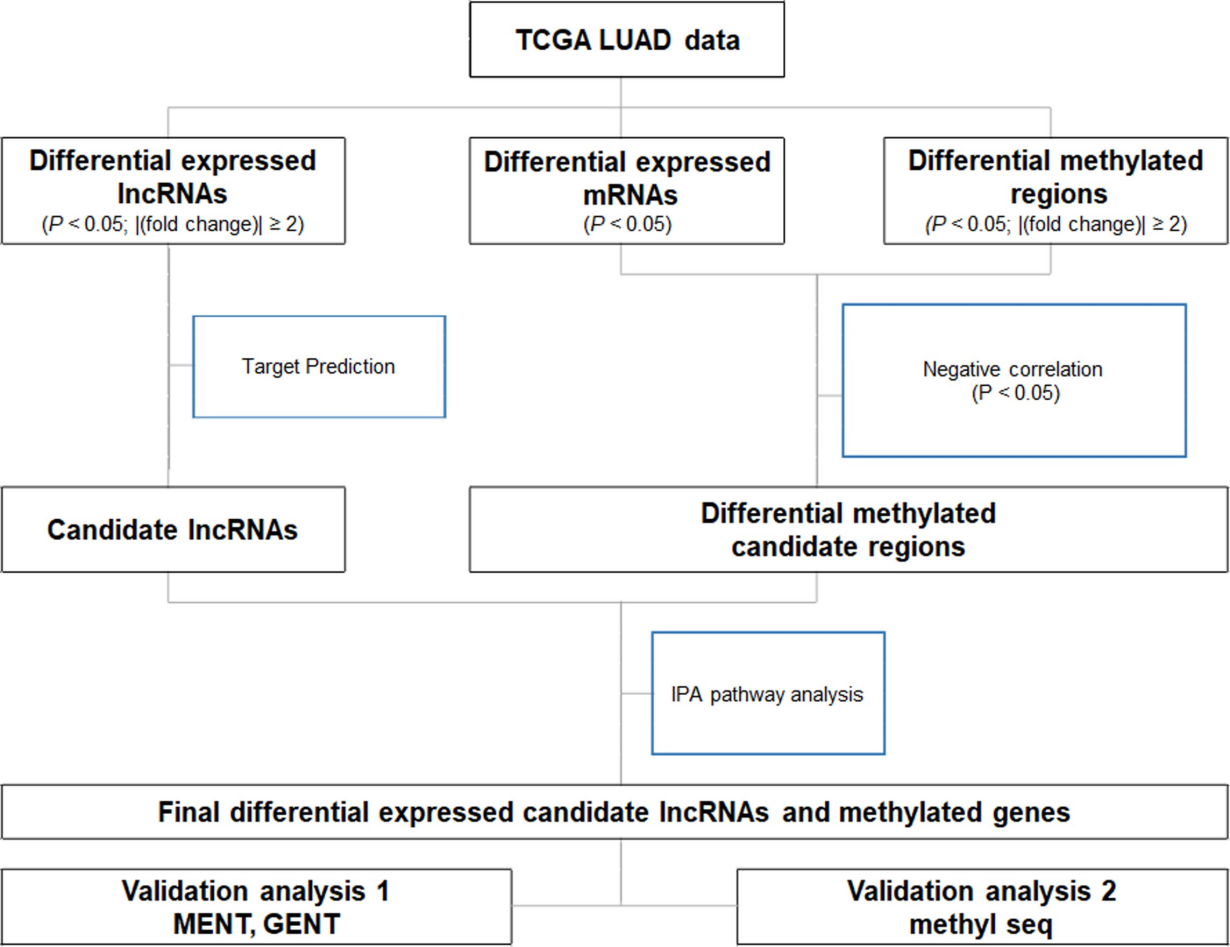
Schematic diagram of the analysis strategy used in this study.

Our analysis strategy is depicted in Fig 1. Differentially expressed genes (DEGs), differentially methylated regions (DMRs), and differentially expressed lncRNAs (DE-lncRNAs) were identified between normal lung, smoker LUAD, and never-smoker LUAD tissues. Ingenuity pathway analysis (IPA) was used to map candidate lncRNAs from the DE-lncRNAs. The matched DEGs and DMRs included those whose change in DNA methylation was inversely correlated with DEG expression (*p* < 0.05).

### Integrated analysis of DE-lncRNAs associated with DMRs

Lists of significant DEGs generated from TCGA data were subjected to IPA using web-based software from Ingenuity Systems® (Qiagen, Redwood City, CA, USA) to produce a gene interaction network. DE-lncRNAs were subjected to biological process enrichment analyses. Functional enrichment analysis was also performed on these networks to understand the significance of the biological functions and/or disease phenotypes of the genes.

### Validation analysis using MENT and GENT

To validate the target DMRs and DE-lncRNAs identified by TCGA data analysis, we utilized the web-accessible public gene expression datasets, Methylation and Expression Database of Normal and Tumor Tissues (MENT; http://mgrc.kribb.re.kr:8080/MENT/) and Gene Expression of Normal and Tumor Tissues (GENT; http://medicalgenome.kribb.re.kr/GENT/). GENT contains the gene expression profiles of 32 types of human cancer tissues and normal tissues generated using an Affymetrix U133A or U133Plus2 microarray platform with consistent data processing [31]. MENT contains DNA methylation and gene expression patterns obtained using an Illumina HumanMethylation27 BeadChip or GoldenGate Methylation Cancer Panel I [32].

### Validation analysis using bisulfite sequencing

Putative genes were validated using bisulfite sequencing in a validation cohort consisting of 76 samples. DNA was quantified using Picogreen (Invitrogen, California, USA) according to the manufacturer’s protocol. Briefly, 1 μg of genomic DNA was bisulfite-converted using EZ DNA Methylation according to manufacturer’s protocol (Zymo Research, California, USA). The regions of interest were amplified by PCR using a KOD-Multi & EPi (Toyobo, Osaka Japan), purified using QIAquick PCR columns (Qiagen, Venlo, Netherlands‎), quantified using Picogreen (Invitrogen), and verified using agarose gel electrophoresis. Libraries were prepared using an Illumina TruSeq Nano DNA sample prep kit (Illumina) according to the manufacturer’s instructions and then quantified by qPCR using a CFX96 Real-Time System (Biorad, California, USA). After normalization, the prepared library was sequenced using a Miseq system (Illumina) with 300 bp paired-end reads.

Potential sequencing adapters and low-quality bases in the raw reads were trimmed using Skewer [33] and the remaining high-quality reads were mapped to the reference genome using BS-seeker2 software [34] with a 10% mis-mapping rate. To compare the CpG methylation profiles of different sample groups, only the CpG site values were selected and the Kruskal-Wallis test was performed.

### Statistical analysis

To identify methylation markers for detecting CS-associated LUAD, we evaluated the distribution of mRNA expression and DNA methylation levels for each CpG site in normal lung, never-smoker LUAD, and smoker LUAD tissues. For candidate DMRs, pairwise comparisons were conducted to identify the genes that best distinguished each group.

## Results

### Identification of DMRs and DE-lncRNAs

**Fig 2:**
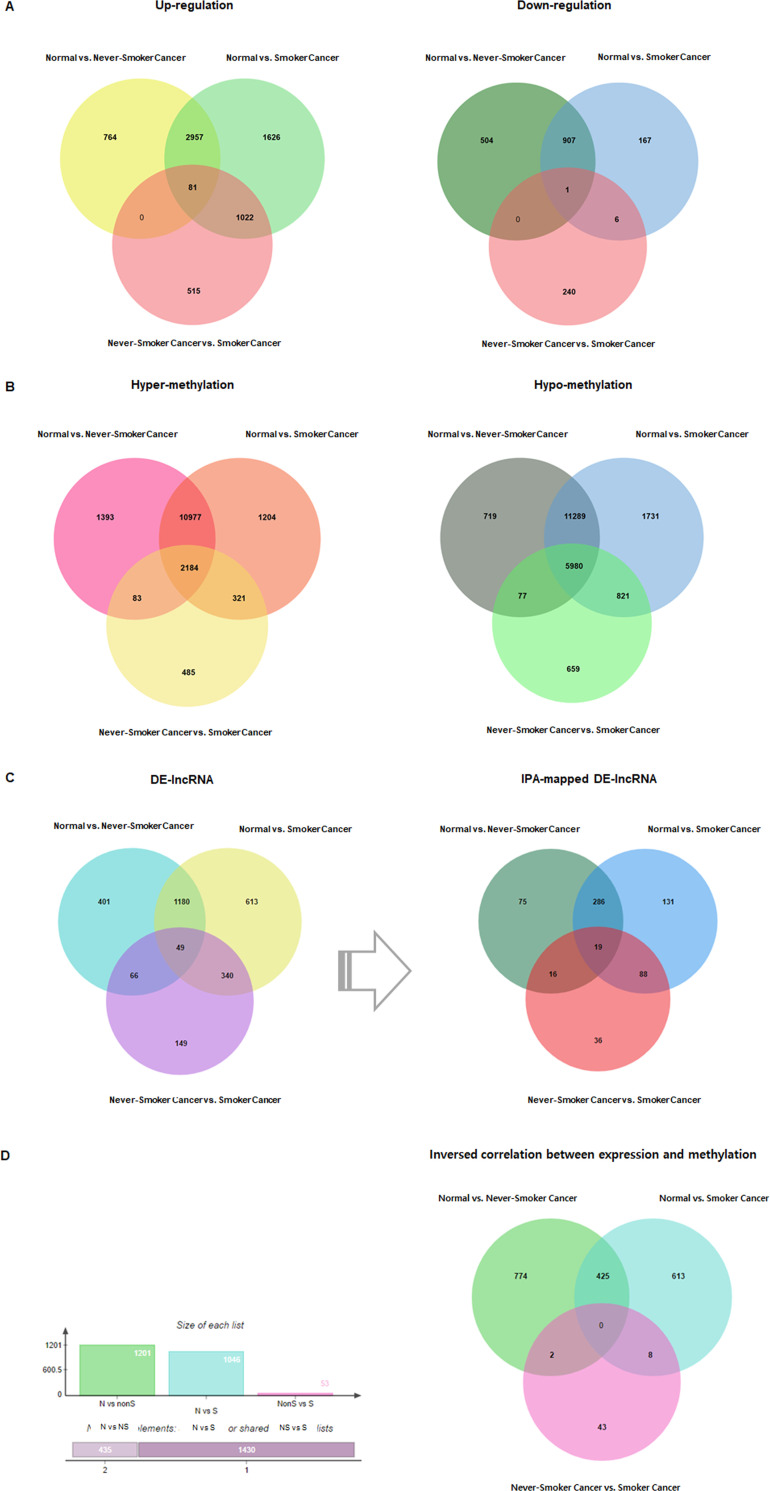
Venn diagrams illustrating the number of differentially expressed transcripts for different pairwise comparisons. (A) Venn diagram of 8,513 differentially expressed genes (DEGs; *p* < 0.05; |(fold change)| ≥ 2). (B) Venn diagram of 24,783 differentially methylated regions (DMRs; *p* < 0.05). (C) Venn diagram of 2,798 differentially expressed lncRNAs (DE-lncRNAs; *p* < 0.05; |(fold change)| ≥ 2) and 1,079 IPA-mapped lncRNAs. (D) Final 1,865 candidate genes displaying inverse correlation.

To investigate the DNA methylation patterns in LUAD related to CS history, we analyzed publicly available Human Methylation 450k TCGA data that measured methylation levels in normal lung and LUAD tissues. The data sets used in this study are summarized in Table 1. Three comparisons were made: 1) normal lung vs. smoker LUAD tissues, 2) normal lung vs. never-smoker LUAD tissues, and 3) never-smoker LUAD vs. smoker LUAD, identifying 8,513 DEGs, 24,783 DMRs, and 2,798 DE-lncRNAs (Fig 2A–2C). Among the 2,798 DE-lncRNAs, 1,079 were mapped by IPA (Fig 2C), while 1,865 differentially methylated candidate genes with negative correlation were identified (Fig 2D) and annotated (S1 Fig) from the DEGs and DMRs.

**Table 1:** Characteristics of TCGA and validation cohorts.

Parameter		TCGA cohort			Validation cohort		
		Smoker (*n* = 159)	Never-smoker (*n* = 25)	Total (*n* = 184)	Smoker (*n* = 24)	Never-smoker (*n* = 52)	Total (*n* = 76)
Gender	Male	70 (89.74%)	8 (10.26%)	78	22 (78.57%)	6 (21.43%)	28
	Female	89 (83.96%)	17 (16.04%)	106	2 (4.17%)	46 (95.83%)	48
Age	Median	67	69	67	69	60	64
Race	White	126 (88.11%)	17 (11.89%)	143	0	0	0
	Asian	1 (100%)	0 (0%)	1	24 (31.58%)	52 (68.42%)	76
	Black or African American	7 (100%)	0 (0%)	7	0	0	0
	Unknown	25 (75.76%)	8 (24.24%)	33	0	0	0
Vital status	Alive	94 (87.85%)	13 (12.15%)	107	22 (32.84%)	45 (67.16%)	67
	Dead	65 (84.42%)	12 (15.58%)	77	2 (22.22%)	7 (77.78%)	9
Tumor stage	I	87 (87%)	13 (13%)	100	3 (27.27%)	8 (72.73%)	11
	II	31 (79.49%)	8 (20.51%)	39	14 (27.45%)	37 (72.55%)	51
	III	34 (89.47%)	4 (10.53%)	38	7 (53.85%)	6 (46.15%)	13
	IV	6 (100%)	0	6	0	1 (100%)	1
	NA	1 (100%)	0	1	0	0	0
*KRAS*	Wild	101 (82.79%)	21 (17.21%)	122	12 (24%)	38 (76%)	50
	Mutant	58 (93.55%)	4 (6.45%)	62	4 (66.67%)	2 (33.33%)	6
	NA	0	0	0	8 (40%)	12 (60%)	20
*EGFR*	Wild	138 (87.34%)	20 (12.66%)	158	18 (48.65%)	19 (51.35%)	37
	Mutant	21 (80.77%)	5 (19.23%)	26	6 (15.38%)	33 (84.62%)	39

TCGA, The Cancer Genome Atlas; NA, not available; *KRAS*, Kirsten rat sarcoma 2 viral oncogene homolog; *EGFR*, Epidermal Growth Factor Receptor.

### Pathway analysis and epigenetically regulated lncRNAs

**Fig 3:**
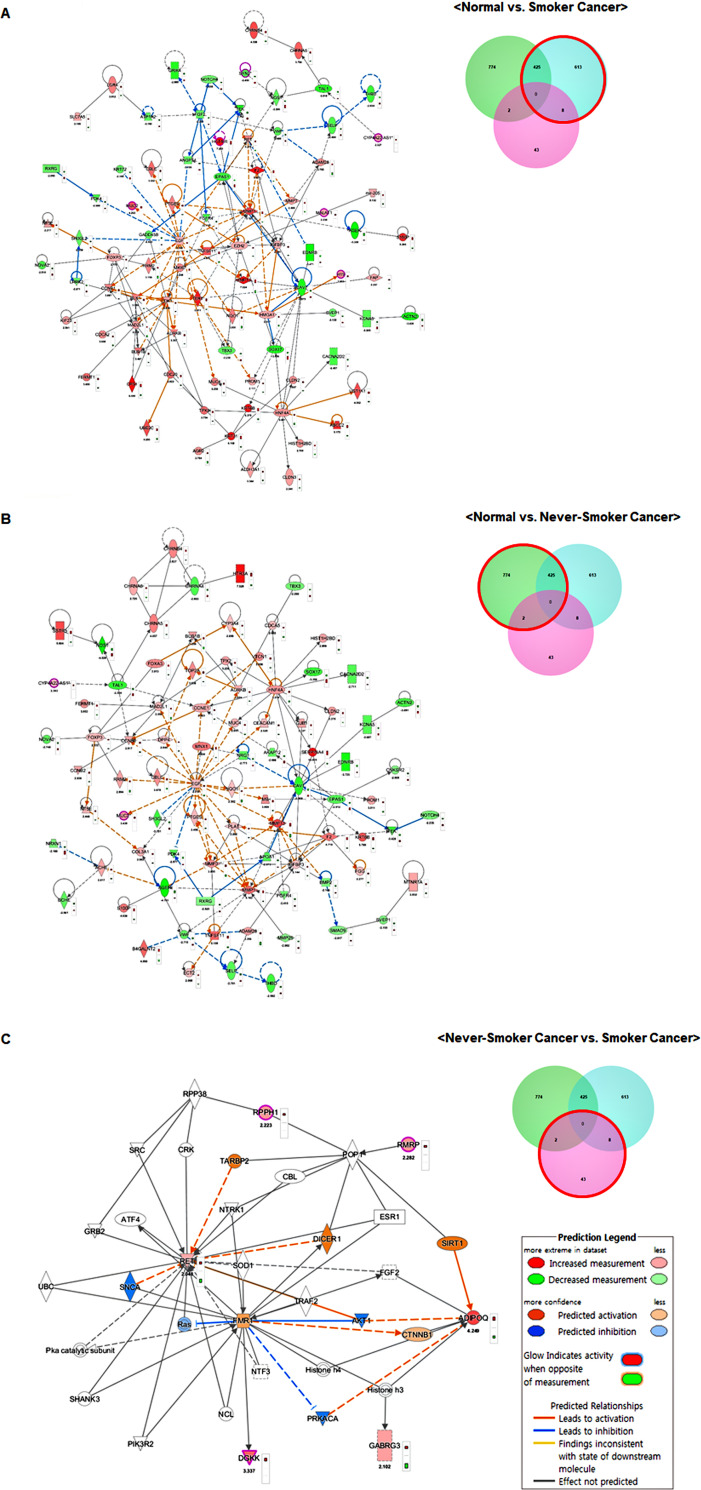
Network of epigenetically regulated genes identified using ingenuity pathway analysis. Each network was displayed graphically with genes or gene products as nodes (different shapes represent different functional classes of gene products) and lines indicating the biological relationships between nodes. The molecular network in normal lung vs. smoker LUAD tissues (A), normal lung vs. never-smoker LUAD tissues (B), and never-smoker LUAD vs. smoker LUAD tissues (C).

A total of 1,865 DMRs were selected as candidate targets for the DE-lncRNAs. To determine the functions of these target genes and their potential network connections, we used IPA to identify the gene networks that may have been affected by these DE-lncRNA target genes (Fig 3). The top ten significant canonical pathways based on the DMRs and DE-lncRNAs are shown in S2 Fig and S1–S3 Tables. Interactions between the DMRs and DE-lncRNAs were predicted using molecular networks based on the IPA molecular database. The most noticeable functional category between never-smoker and smoker LUAD tissues was the lipopolysaccharide (LPS)/IL-1 mediated inhibition of retinoid X receptors (RXR) function.

A total of 86 candidate genes including six lncRNAs were identified by comparing smoker LUAD and normal tissues. Of the 43 candidate genes identified by comparing never-smoker LUAD and smoker LUAD tissues, 13 also displayed differences when compared to normal tissues. Although the majority of top functional pathways and related molecules overlapped when comparing 1) normal lung vs. smoker LUAD tissues and 2) normal lung vs. never-smoker LUAD tissues, notable differences were observed when comparing smoker LUAD and never-smoker LUAD tissues, including the LPS/IL-1-mediated inhibition of RXR function and nicotine degradation III.

We identified six lncRNAs that were significantly differentially expressed in normal lung vs. smoker LUAD tissues (Table 2). Among these, *HOTAIR*, *Synapsin II* (*SYN2)*, *MALAT1*, and *H19* were uniquely expressed in smoker LUAD tissues, while *CYP4A22 antisense RNA 1 (CYP4A22-AS1)* and *Lnc-MUC2-1* expression overlapped in normal lung vs. never-smoker LUAD tissues. In addition, four lncRNAs were significantly differentially expressed in never-smoker LUAD vs. smoker LUAD tissues (Table 3). These findings suggest that the lncRNAs may be involved in CS-induced epigenetic alterations in patients with LUAD.

**Table 2:** Epigenetically regulated lncRNAs in normal lung vs. smoker LUAD tissues.

Gene ID	Regulation	FDR	Chr.	Class	Strand
CYP4A22-AS1*	Up	7.E-09	1	Intergenic, antisense	-
Lnc-MUC2-1*	Up	2.E-04	11	Intergenic	+
*HOTAIR*	Up	2.E-07	12	Intergenic, antisense	-
*SYN2*	Down	9.E-14	3	Sense-overlapping	+
*MALAT1*	Up	9.E-04	11	Antisense	+
*H19*	Up	1.E-03	11	Intergenic	-

FDR, false discovery rate; Chr., chromosome.

* LncRNAs whose expression overlapped with normal lung vs. never-smoker LUAD tissues.

**Table 3:** Gene validation between never-smoker LUAD and smoker LUAD tissues.

Gene	Log fold change (expression)	*P* value (methylation)	LncRNA	GENT and MENT matching
SBSN	6.4	9.E-14		O
RP11-474D1.3	6.0	4.E-02	O	
MSLNL	2.5	9.E-03		
SLC3A1	2.8	5.E-02		O
MSMB	3.8	1.E-04		O
ATP11AUN	3.6	1.E-03	O	
TCP11	2.3	6.E-06		
KRTDAP	3.4	8.E-07		
ALDH3A1	2.1	4.E-02		
ADAM6	4.3	2.E-04	O	O
CYP4F3	2.1	2.E-09		
CTC-518B2.9	2.0	9.E-07	O	
ADIPOQ	2.6	2.E-03		O

*SBSN*, suprabasin; *MSLNL*, mesothelin like; *SLC3A1*, solute carrier family 3 member 1; *MSMB*, microseminoprotein beta; *ATP11AUN*, *ATP11A* upstream neighbor; *TCP11*, t-complex 11; *KRTDAP*, keratinocyte differentiation associated protein; *ALDH3A1*, aldehyde dehydrogenase 3 family member A1; *ADAM6*, *ADAM* metallopeptidase domain 6; *CYP4F3*, cytochrome P450 family 4 subfamily F member 3; *ADIPOQ*, adiponectin, *C1Q* and collagen domain containing.

### Validation of gene expression profiles using MENT and GENT

First, we investigated the expression and methylation levels of 86 genes in normal lung vs. smoker LUAD tissues and 13 genes in never-smoker LUAD vs. smoker LUAD tissues using the GENT and MENT databases. When comparing the 86 genes in smoker LUAD and normal lung tissues, seven up-regulated and six down-regulated genes were inversely correlated with methylation (Table 4), while five of the 13 genes were inversely correlated with methylation in smoker LUAD compared to never-smoker LUAD tissues (Table 3).

**Table 4:** DEGs between normal lung and smoker LUAD tissues with inverse correlation in the GENT and MENT databases.

Gene	Regulation	Chr.	Start (bp)	End (bp)	Size (bases)
*AURKB*	Up	17	8,204,731	8,210,767	6,046
*CAV1*	Down	7	116,524,785	116,561,185	36,401
*EGF*	Up	4	109,912,883	110,012,962	100,080
*KCNA5*	Down	12	5,043,919	5,046,788	2,870
*MMP13*	Up	11	102,942,992	102,955,734	12,743
*TEK*	Down	9	27,109,141	27,230,178	121,038
*AGR2*	Up	7	16,791,811	16,833,433	41,623
*CCNB1*	Up	5	69,167,010	69,178,245	11,236
*FGF2*	Down	4	122,826,708	122,898,236	71,529
*HMGA1*	Up	6	34,236,800	34,246,231	9,432
*HNF4A*	Up	20	44,355,700	44,434,596	78,897
*SOX17*	Down	8	54,457,935	54,460,896	2,962
*TAL1*	Down	1	47,216,290	47,232,373	16,084

Chr., chromosome; bp, base pair; *AURKB*, aurora kinase B; *CAV1*, caveolin 1; *EGF*, epidermal growth factor; *KCNA5*, potassium voltage-gated channel subfamily A member 5; *MMP13*, matrix metallopeptidase 13; *TEK*, *TEK* receptor tyrosine kinase; *AGR2*, anterior gradient 2; *CCNB1*, cyclin B1; *FGF2*, fibroblast growth factor 2; *HMGA1*, high mobility group AT-hook 1; *HNF4A*, hepatocyte nuclear factor 4 alpha; *SOX17*, *SRY*-box transcription factor 17; *TAL1*, *TAL* bHLH transcription factor 1.

### Validation of gene expression profiles using bisulfite sequencing

**Fig 4:**
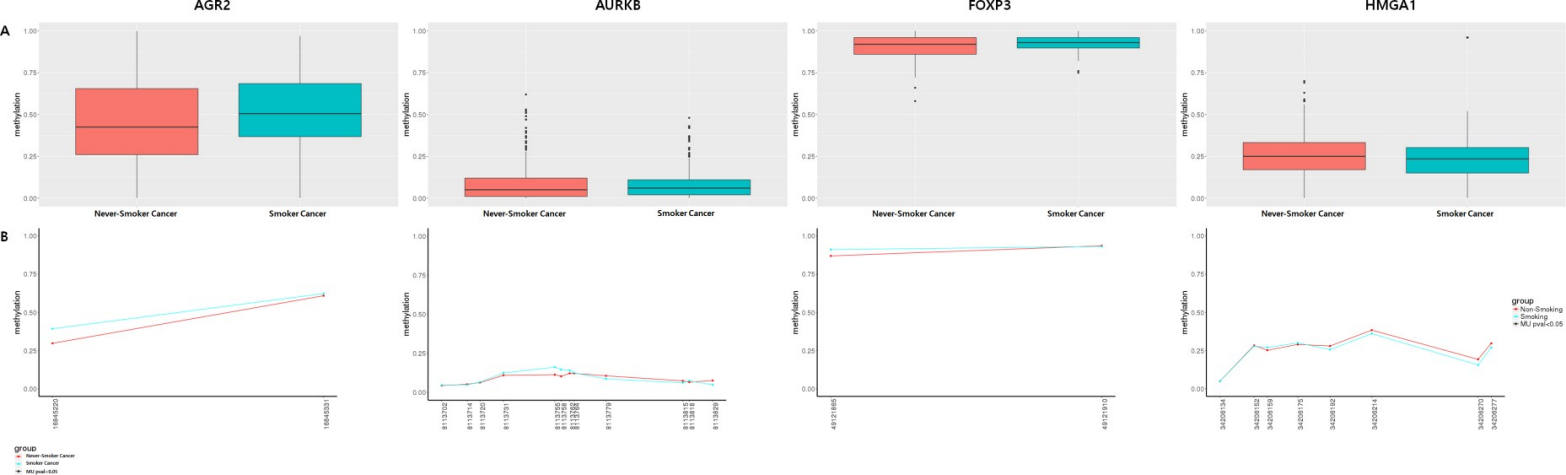
Comparison of methylation levels in never-smoker and smoker LUAD tissues. (A) Box plot showing the average methylation score. (B) Methylation profile plot showing differentially methylated CpG sites (DMCpG) by absolute position (red, never-smoker LUAD; blue, smoker LUAD).

Finally, we performed bisulfite sequencing on *ADAM Metallopeptidase Domain 6 (ADAM6)*, *Anterior Gradient 2 (AGR2)*, *Aurora Kinase B (AURKB)*, *budding uninhibited by benzimidazoles 1 homolog beta (BUB1B)*, *Caveolin 1 (CAV1)*, *Cyclin B1 (CCNB1)*, *forkhead box P3(FOXP3)*, *high mobility group AT-hook 1(HMGA1)*, *Matrix metallopeptidase 13(MMP13)*, and *Suprabasin (SBSN)* using LUAD samples (S3 and S4 Figs, and Table 1). Four CpG sites in *AGR2*, *AURKB*, *FOXP3*, and *HMGA1* displayed borderline significance (Fig 4 and Table 5).

**Table 5:** DNA methylation differences for candidate genes in 52 never-smoker and 24 smoker LUAD tissues.

Gene	Chromosome	CpG site	Mean beta value		*P* value
			Never-smoker	Smoker	
*ADAM6*	14	106,438, 118	0.28	0.25	0.38
		106,438,144	0.30	0.26	0.33
		106,438,159	0.88	0.84	0.14
		106,438,164	0.34	0.33	0.75
		106,438,176	0.38	0.37	0.67
		106,438,219	0.79	0.81	0.68
		106,438,221	0.80	0.78	0.48
		106,438,231	0.35	0.32	0.55
		106,438,251	0.82	0.81	0.94
*AGR2*	7	16,845,220	0.30	0.39	0.07
		16,845,331	0.61	0.62	0.83
*AURKB*	17	8,113,702	0.04	0.05	0.22
		8,113,714	0.05	0.05	0.69
		8,113,720	0.06	0.07	0.88
		8,113,731	0.11	0.12	0.51
		8,113,755	0.11	0.16	0.14
		8,113,758	0.10	0.15	0.06
		8,113,762	0.12	0.14	0.70
		8,113,764	0.12	0.13	0.85
		8,113,779	0.11	0.09	0.37
		8,113,815	0.07	0.06	0.75
		8,113,818	0.07	0.07	0.76
		8,113,829	0.08	0.05	0.45
*BUB1B*	15	40,453,006	0.08	0.09	0.50
		40,453,010	0.10	0.08	0.62
		40,453,023	0.08	0.08	0.96
		40,453,029	0.13	0.08	0.80
		40,453,034	0.14	0.10	0.73
		40,453,036	0.12	0.10	0.84
		40,453,091	0.13	0.14	0.72
		40,453,131	0.09	0.12	0.26
		40,453,133	0.08	0.13	1.00
		40,453,139	0.04	0.05	0.46
		40,453,141	0.01	0.04	0.24
*CAV1*	7	116,164,422	0.26	0.23	0.62
		116,164,436	0.26	0.24	0.55
		116,164,533	0.23	0.16	0.24
*CCNB1*	5	68,462,676	0.07	0.06	0.83
		68,462,690	0.08	0.08	0.20
		68,462,711	0.10	0.10	0.36
		68,462,714	0.10	0.12	0.09
		68,462,743	0.12	0.12	0.57
		68,462,756	0.10	0.09	0.93
		68,462,762	0.09	0.10	0.16
		68,462,768	0.09	0.08	0.99
		68,462,783	0.05	0.06	0.33
*FOXP3*	X	49,121,865	0.87	0.91	0.08
		49,121,910	0.94	0.93	0.49
*HMGA1*	6	34,206,134	0.05	0.05	0.95
		34,206,152	0.28	0.28	0.36
		34,206,159	0.25	0.27	0.96
		34,206,175	0.29	0.30	0.80
		34,206,192	0.28	0.26	0.09
		34,206,214	0.38	0.36	0.30
		34,206,270	0.19	0.16	0.13
		34,206,277	0.30	0.27	0.06
*MMP13*	11	102,826,680	0.59	0.58	0.90
*SBSN*	19	36,019,108	0.93	0.93	0.84
		36,019,115	0.89	0.86	0.36
		36,019,162	0.76	0.73	0.47
		36,019,170	0.76	0.74	0.61
		36,019,198	0.53	0.52	0.67

*ADAM6*, *ADAM* metallopeptidase domain 6; *AGR2*, anterior gradient 2; *AURKB*, aurora kinase B; *BUB1B*, budding uninhibited by benzimidazoles 1 homolog beta; *CAV1*, caveolin 1; *CCNB1*, Cyclin B1; *FOXP3*, forkhead box P3; *HMGA1*, high mobility group AT-hook 1; *MMP13*, matrix metallopeptidase 13; *SBSN*, suprabasin.

## Discussion

In this study, we integrated DNA methylation, lncRNA expression, and mRNA expression profiles from TCGA, identified biomarker candidates, and validated our findings using public datasets from the GENT and MENT databases as well as an external cohort. Together, our findings contribute toward our understanding of the interplay between lncRNAs and DNA methylation and provide a map of the epigenetic landscape of lung cancer. In addition, this study is the first to reveal the potential role of lncRNAs in CS-associated epigenetic regulation in LUAD.

Based on the findings of previous reports, we expected to find a significant difference in epigenetic alterations between smoker and never-smoker LUAD tissues. Consistently, we found differences in regulatory genes and identified ten lncRNAs: *HOTAIR*, *SYN2*, *MALAT1*, and *H19* in smoker LUAD vs. normal lung tissues, *CYP4A22-AS1* and *Lnc-MUC2-1* in both smoker and never-smoker LUAD vs. normal lung tissues, and *RP11-474D1*.*3*, *ATP11AUN*, *ADAM6*, and *CTC-518B2*.*9* in smoker vs. never-smoker LUAD tissues. And the main biochemical functions revealed by our analyses were inconsistent. For the differentially expressed transcripts in smoker LUAD tissues, the major enriched pathways were the coagulation system, granulocyte adhesion, and diapedesis, whereas the primary pathways for transcripts in never-smoker LUAD tissues were axonal guidance signaling and atherosclerosis signaling. GENT and MENT analysis in these two tissue types revealed five genes, including *ADAM6* and *SBSN*, that displayed an inverse correlation between gene expression and methylation levels.

Until now, only a small number of lncRNAs have been identified in CS-associated lung cancer, several of which have been suggested as possible diagnostic and prognostic biomarkers. For instance, the novel lncRNA *SCAL1* was reported to be overexpressed in lung cancer cell lines as a result of CS-induced oxidative stress [28]. Moreover, other studies have suggested that *SCAL1* expression may be regulated by *Nuclear Factor Erythroid 2-Related Factor* (*NRF2*) and that it may mediate cytoprotective functions against CS-induced toxicity [28,35,36]. *HOTAIR* expression is also significantly up-regulated in lung cancer and correlates with metastasis and poor prognosis [37–42], furthermore, Liu et al. found that *HOTAIR* up-regulation contributes toward CS-induced malignant transformation mediated by *STAT3* signaling [27]. Elevated *H19* expression has also been detected in lung cancer [43–45] and its overexpression has been observed in smokers compared to never-smokers [46]. One *in vitro* study investigated CS-induced increases in *H19* expression and attributed the increase to the mono-allelic up-regulation of normally expressed alleles [29]. In addition, high *MALAT1* expression has been identified in metastatic lung cancer and was shown to be an independent prognostic indicator of early-stage tumors [47], and further studies have reported *MALAT1* to be involved in CS-induced epithelial-mesenchymal transition and malignant transformation via *Enhancer of Zeste Homolog 2* (*EZH2*), a well-known epigenetic regulator [30,48–50].

The majority of previous studies have investigated possible molecular mechanisms and novel biomarkers associated with epigenetic changes using wet laboratory experiments; however, integrated analysis based on bioinformatics methods and prediction may be more efficient for translational research, but such studies are currently lacking. Since a single lncRNA targets numerous transcripts and a single transcript is also regulated by numerous lncRNAs, lncRNAs can induce various functional pathways and have complicated regulatory networks. Consequently, it is difficult to rank candidate lncRNAs during the experimental design and validation processes when exploring the functions of lncRNAs. Considering this complexity, integrating datasets could be an effective and promising approach to infer functional networks and verify potential targets. Indeed, utilizing datasets and developing computational models to predict lncRNA associations and functional annotations are currently emerging fields [51,52].

In this study, we used bioinformatics methods to identify potential targets and their functions that may play critical roles in the control of lung cancer. The most significantly different functional category between never-smoker and smoker LUAD tissues was the LPS/IL-1 mediated inhibition of RXR function. RXRs are retinoid receptors that play a crucial role in regulating the growth and differentiation of normal and tumor cells [53], while retinoids are known for their role as epigenetic modifiers [54]. Su Man et al. previously observed that the effect of RXR gene methylation on prognosis differed significantly between never-smokers and smokers, and suggested that methylation-associated RXR gene down-regulation may play different roles in lung carcinogenesis depending on smoking status [55,56]. To some extent, our findings are consistent with those of this previous study and emphasize the importance of the identified molecules.

Besides the well-known lncRNAs mentioned earlier, we identified other significant DE-lncRNAs in this study, including *SYN2*, *RP11-474D1*.*3*, *ATP11AUN*, *ADAM6*, and *CTC-518B2*.*9*; however, their molecular mechanisms in CS-induced LUAD remain largely unknown. Since our findings suggest possible associations between these lncRNAs and lung cancer, we believe that their specific functions should be characterized experimentally.

Despite the important findings we have described, this study had several limitations. Firstly, the patients included in TCGA database were mostly white, whereas the samples used for bisulfite sequencing validation were derived from Korean patients. Since genomic mutations such as epigenetic changes can differ between races [57–59], these racial disparities may have affected our results. Secondly, the mechanisms of epigenetic regulation by lncRNAs in CS-induced lung cancer development were not confirmed as this can be challenging; however, experimental strategies such as genetically manipulating the lncRNA locus or deleting of the full-length lncRNA locus or its promoter sequence *in vivo* could provide further functional information. Thirdly, we assumed a negative correlation between DEGs and DMRs when searching for candidate genes since methylation levels are generally negatively correlated with the expression levels of nearby genes [60]. However, when gene expression is tightly regulated or 5-hydroxymethylcytosine (5hMc) activates transcription, this trend is reversed [61,62]. Since we excluded the possibility of this effect in this study, more comprehensive algorithms will be required to determine the diversity of crosstalk between methylation, expression, and regulation elements. Lastly, we did not analyze any other environmental factor except for smoking due to a lack of information regarding the occupation or dwelling of the patients whose samples were used in this study. Recent reports have strongly associated exposure to outdoor particulate matter (PM10) [63,64] or indoor high temperature cooking oil fumes [65] with lung cancer; therefore, controlling such factors that affect epigenetic alterations would provide more accurate results.

In summary, we identified dysregulated lncRNAs that mediate DNA methylation in CS-associated LUAD using integrated analyses. Although the roles of these lncRNAs in LUAD are currently unclear, our findings suggest that their molecular mechanisms warrant further investigation. Therefore, the continued investigation of the lncRNAs identified in this study will aid the development of guidelines to assess individual risk for lung cancer and its prevention.

## Supporting information

S1 Fig Annotation of differentially methylated candidate genes. (TIF)

S2 Fig Canonical pathways identified from the functional annotation of candidate DMRs and lncRNAs. (A) Comparison between normal lung and smoker LUAD tissues. (B) Comparison between normal lung and never-smoker LUAD tissues. (C) Comparison between never-smoker LUAD and smoker LUAD tissues.
(TIF)

S3 Fig Comparison of methylation levels between never-smoker and smoker LUAD tissues. (A) Box plot showing the average methylation score. (B) Methylation profile plot showing differentially methylated CpG sites (DMCpG) by absolute position (red, never-smoker LUAD; blue, smoker LUAD).
(TIF)

S4 Fig Denogram of methylation profiles revealed by bisulfite sequencing. (red, never-smoker LUAD; blue, smoker LUAD).
(TIF)

S1 Table Canonical pathways identified from the functional annotation of normal lung and smoker LUAD tissues. (PDF)

S2 Table Canonical pathways identified from the functional annotation of normal lung and never-smoker LUAD tissues. (PDF)

S3 Table Canonical pathways identified from the functional annotation of never-smoker and smoker LUAD tissues. (PDF)
