# Supplementary figures and images for: Landscape of epigenetically regulated lncRNAs and DNA methylation in smokers with lung adenocarcinoma
Source: PLoS One. 2021 Mar 8;16(3):e0247928. doi: 10.1371/journal.pone.0247928 (PMC7939300; doi:10.1371/journal.pone.0247928)

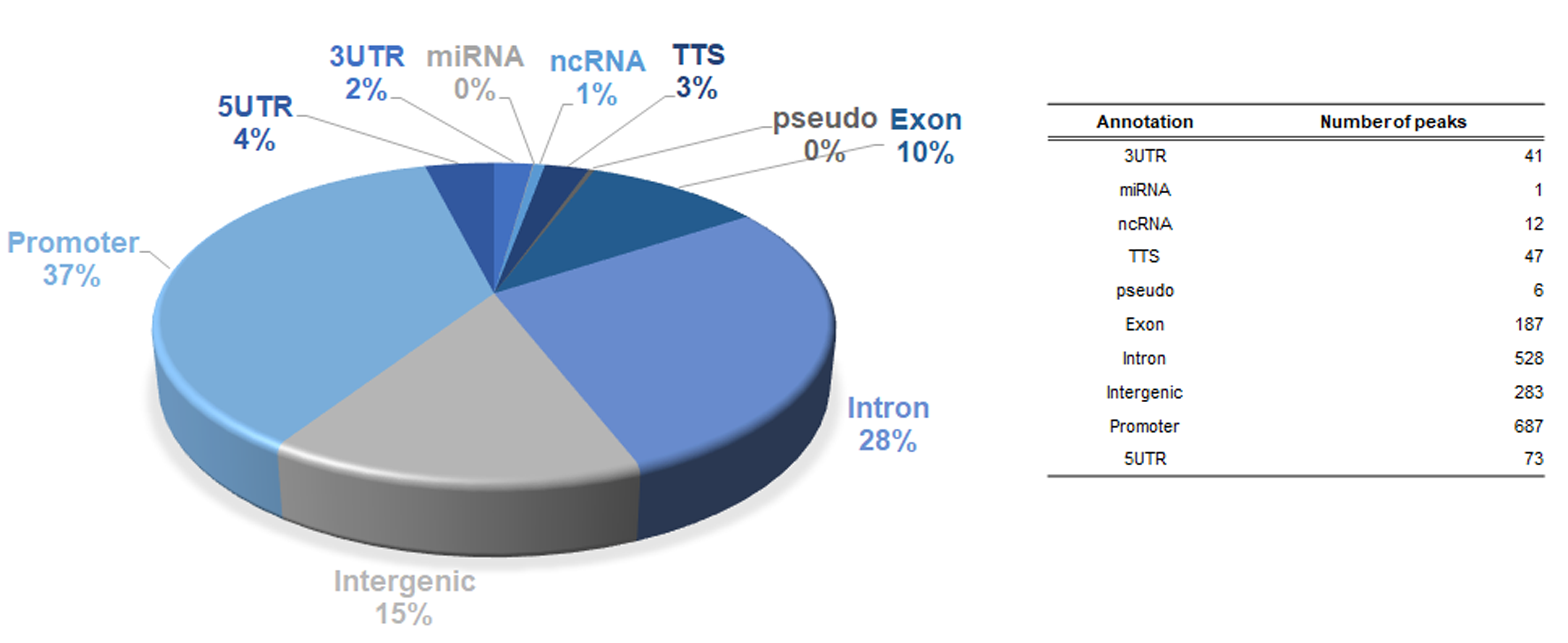

Supplement: S1 Fig — (TIF) [file pone.0247928.s001.tif]

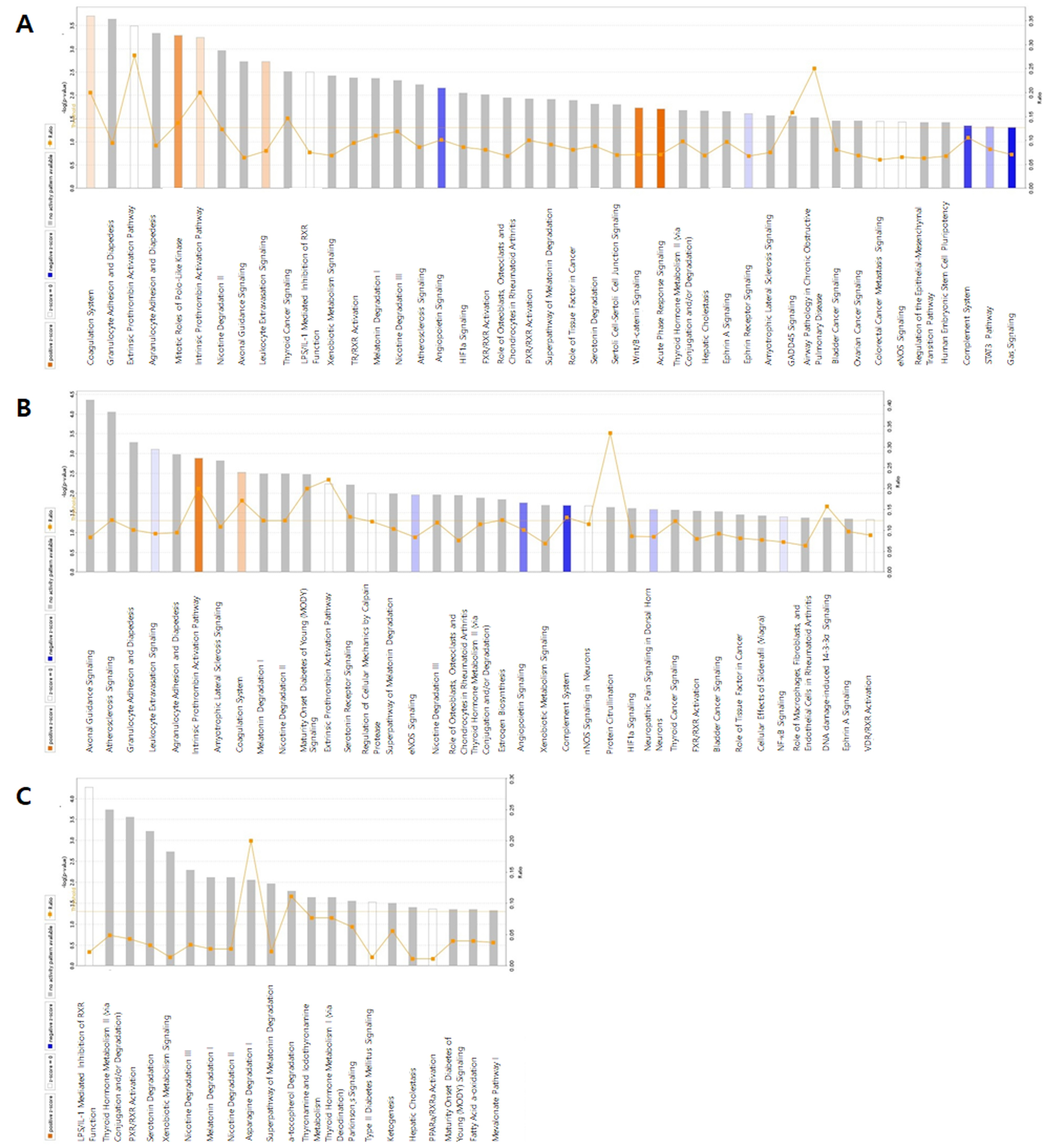

Supplement: S2 Fig — (A) Comparison between normal lung and smoker LUAD tissues. (B) Comparison between normal lung and never-smoker LUAD tissues. (C) Comparison between never-smoker LUAD and smoker LUAD tissues. (TIF) [file pone.0247928.s002.tif]

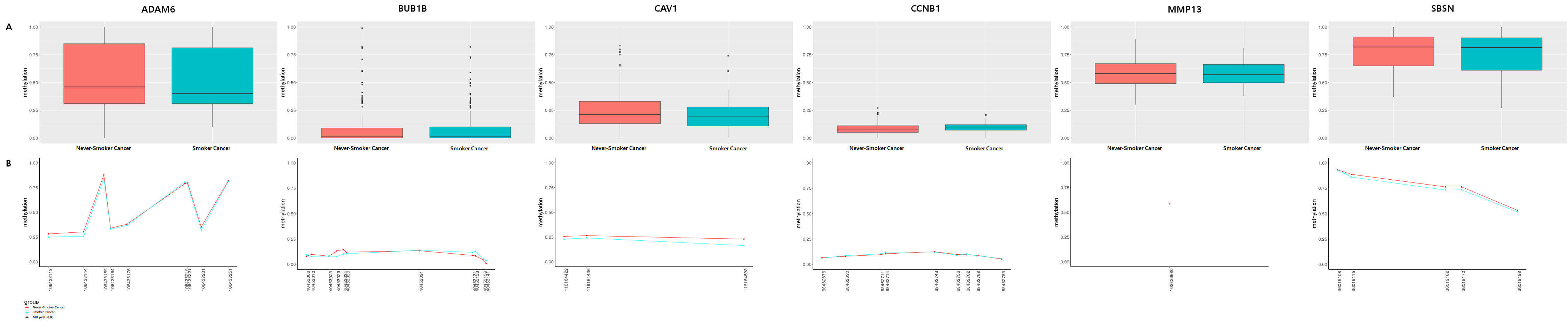

Supplement: S3 Fig — (A) Box plot showing the average methylation score. (B) Methylation profile plot showing differentially methylated CpG sites (DMCpG) by absolute position (red, never-smoker LUAD; blue, smoker LUAD). (TIF) [file pone.0247928.s003.tif]

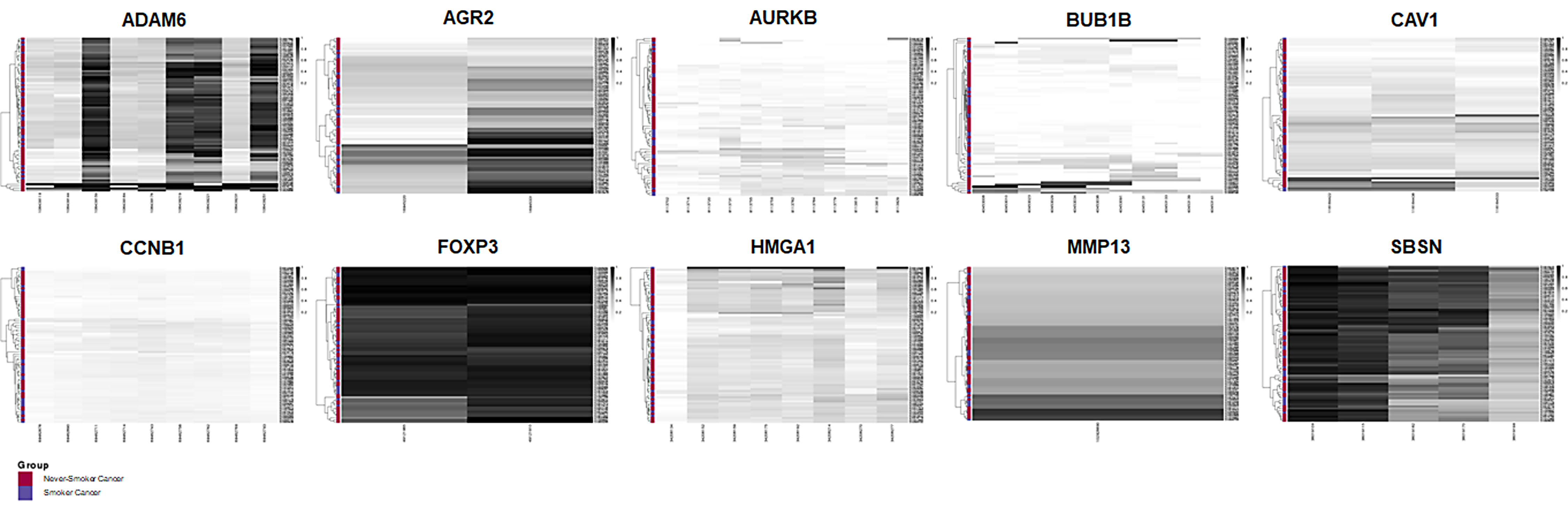

Supplement: S4 Fig — (red, never-smoker LUAD; blue, smoker LUAD). (TIF) [file pone.0247928.s004.tif]
