# Supplementary material for: Landscape of epigenetically regulated lncRNAs and DNA methylation in smokers with lung adenocarcinoma
Source: PLoS One. 2021 Mar 8;16(3):e0247928. doi: 10.1371/journal.pone.0247928 (PMC7939300; doi:10.1371/journal.pone.0247928)
Supplement: S1 Table — (PDF) [file pone.0247928.s005.pdf]

Ingenuity Canonical Pathways	-Log(*P* value)	Molecules
Coagulation System	3.71	F11,F10,VWF,F7,FGA,THBD,F2
Granulocyte Adhesion and Diapedesis	3.64	CLDN10,MMP7,CLDN18,IL36A, CCL24,MMP13,CLDN6,FPR1,CXCL3,CLDN5,SELP,CDH5,IL36RN,CCL25,CLDN2,MMP1,CLDN3
Extrinsic Prothrombin Activation Pathway	3.49	F10,F7,FGA,THBD,F2
Agranulocyte Adhesion and Diapedesis	3.34	AOC3,CLDN10,MMP7,CLDN18, IL36A,CCL24,MMP13,CLDN6,CXCL3,CLDN5,CDH5,SELP,IL36RN,CCL25,CLDN2,MMP1,CLDN3
Mitotic Roles of Polo-Like Kinase	3.28	KIF23,CDC25C,ESPL1,CDC20,PTTG1,PRC1,PLK1,CDK1,CCNB1
Intrinsic Prothrombin Activation Pathway	3.25	F11,COL1A1,F10,FGA,THBD,F2
Nicotine Degradation II	2.96	CYP3A7,CYP2F1,FMO2,UGT2B10,LARGE2,INMT,UGT1A1,UGT1A7, UGT2B15
Axonal Guidance Signaling	2.73	SLIT3,ADAMTS8,MMP7,BDNF, SEMA6A,EGF,MMP13,EPHA8, WNT7A,ADAM28,KL,FGFR4,DCC,TUBA3C/TUBA3D,PLXNB3, GNG4,SEMA3G,ARHGEF15,SEMA5A,EFNA3,SLIT2,HHIP,EFNA4,EPHA10,WNT3A,MAG,WNT10A,NTRK3,PAK5

Leukocyte Extravasation Signaling	2.72	CLDN10,ARHGAP6,MMP7,ACTN2,CLDN18,JAM2,MMP13,RAPGEF4,CLDN6,CLDN5,CDH5,KL,FGFR4,CLDN2,DLC1,MMP1,CLDN3
Thyroid Cancer Signaling	2.51	RXRG,GDNF,BDNF,NTRK3,RET,HNF1A
