# Supplementary material for: Landscape of epigenetically regulated lncRNAs and DNA methylation in smokers with lung adenocarcinoma
Source: PLoS One. 2021 Mar 8;16(3):e0247928. doi: 10.1371/journal.pone.0247928 (PMC7939300; doi:10.1371/journal.pone.0247928)
Supplement: S2 Table — (PDF) [file pone.0247928.s006.pdf]

| Ingenuity Canonical Pathways | -Log(*P* value) | Molecules |
|---|---|---|
| Axonal Guidance Signaling | 4.36 | SLIT3,ADAMTS8,MMP3,WNT3,EPHB2,BDNF,BMP2,SEMA6A,NOTUM,EGF,MMP13,ADAM8,NTNG1,SEMA6D,EPHA8,ADAM28,KL,FGFR4,DCC,TUBA3C/TUBA3D,SEMA3B,PLXNB3,GNG4,EFNA2,SEMA3G,PRKCQ,ADAMTS1,ARHGEF15,SEMA5A,SLIT2,HHIP,EFNA4,EPHA10,WNT3A,MAG,NTRK3,LINGO1,PAK5 |
| Atherosclerosis Signaling | 4.05 | LPA,MMP3,CD36,IL36A,MMP13,IL37,COL1A1,APOA1,PLA2G2F,SELP,LPL,PLA2G4F,MMP1,PAFAH1B3,COL3A1,APOC3 |
| Granulocyte Adhesion and Diapedesis | 3.28 | MMP3,CCL23,CLDN18,IL36A,CCL24,MMP13,IL37,MMP25,CLDN6,FPR1,CLDN5,SELP,CDH5,CCL25,CLDN14,CLDN2,CLDN9,MMP1 |
| Leukocyte Extravasation Signaling | 3.11 | ARHGAP6,PRKCQ,MMP3,ACTN2,CLDN18,JAM2,MMP13,BMX,RAPGEF4,MMP25,CLDN6,CLDN5,CDH5,KL,FGFR4,CLDN14,CLDN2,CLDN9,DLC1,MMP1 |
| Agranulocyte Adhesion and Diapedesis | 2.97 | AOC3,MMP3,CCL23,CLDN18,IL36A,CCL24,MMP13,IL37,MMP25,CLDN6,CLDN5,SELP,CDH5,CCL25,CLDN2,CLDN14,CLDN9,MMP1 |
| Intrinsic Prothrombin Activation Pathway | 2.88 | COL1A1,PROC,THBD,FGG,F2,COL3A1 |
| Amyotrophic Lateral Sclerosis Signaling | 2.81 | CAPN5,NOS1,GRIN1,CACNA1S,GDNF,GRIA1,CASP12,CAPN8,CACNA1E,HE |

| | | |
|---|---|---|
| | | CW1,GRIK4,KL,FGFR4 |
| Coagulation System | 2.52 | PROC,VWF,THBD,FGG,F2,PLAT |
| Melatonin Degradation I | 2.48 | SULT1C4,SULT1C2,CYP3A4,CYP2C18, UGT2B10,LARGE2,CYP2B6,UGT1A7, UGT2B15 |
| Nicotine Degradation II | 2.48 | FMO2,CYP3A4,CYP2C18,UGT2B10,LARGE2,CYP2B6,INMT,UGT1A7, UGT2B15 |
