# Supplementary material for: Landscape of epigenetically regulated lncRNAs and DNA methylation in smokers with lung adenocarcinoma
Source: PLoS One. 2021 Mar 8;16(3):e0247928. doi: 10.1371/journal.pone.0247928 (PMC7939300; doi:10.1371/journal.pone.0247928)
Supplement: S3 Table — (PDF) [file pone.0247928.s007.pdf]

Ingenuity Canonical Pathways	-Log(*P* value)	Molecules
LPS/IL-1 Mediated Inhibition of RXR Function	4.26	IL1R2,ABCC2,FABP7,HMGCS2,ALDH3A1
Thyroid Hormone Metabolism II (via Conjugation and/or Degradation)	3.73	UGT3A1,DIO1,UGT1A7
PXR/RXR Activation	3.56	ABCC2,HMGCS2,UGT1A7
Serotonin Degradation	3.22	UGT3A1,ALDH3A1,UGT1A7
Xenobiotic Metabolism Signaling	2.73	CES1,ABCC2,ALDH3A1,UGT1A7
Nicotine Degradation III	2.29	UGT3A1,UGT1A7
Melatonin Degradation I	2.11	UGT3A1,UGT1A7
Nicotine Degradation II	2.11	UGT3A1,UGT1A7
Asparagine Degradation I	2.04	ASPG
Superpathway of Melatonin Degradation	1.96	UGT3A1,UGT1A7

LPS, lipopolysaccharide; RXR, retinoid X receptors; PXR, pregnane X receptor
